# Surveying alignment-free features for Ortholog detection in related yeast proteomes by using supervised big data classifiers

**DOI:** 10.1186/s12859-018-2148-8

**Published:** 2018-05-03

**Authors:** Deborah Galpert, Alberto Fernández, Francisco Herrera, Agostinho Antunes, Reinaldo Molina-Ruiz, Guillermin Agüero-Chapin

**Affiliations:** 1 Departamento de Ciencia de la Computación, Universidad Central ¨Marta Abreu¨ de Las Villas (UCLV), 54830 Santa Clara, Cuba; 2 Department of Computer Science and Artificial Intelligence, Research Center on Information and Communications Technology (CITIC-UGR), University of Granada, 18071 Granada, Spain; 3 CIIMAR/CIMAR, Centro Interdisciplinar de Investigação Marinha e Ambiental, Universidade do Porto, Terminal de Cruzeiros do Porto de Leixões, Av. General Norton de Matos s/n 4450-208 Matosinhos, Porto, Portugal; 4 Departamento de Biologia, Faculdade de Ciências, Universidade do Porto, Rua do Campo Alegre, 4169-007 Porto, Portugal; 5 Centro de Bioactivos Químicos (CBQ), Universidad Central ¨Marta Abreu¨ de Las Villas (UCLV), 54830 Santa Clara, Cuba

**Keywords:** Ortholog detection, Pairwise protein similarity measures, Big data, Supervised classification, Imbalance data

## Abstract

**Background:**

The development of new ortholog detection algorithms and the improvement of existing ones are of major importance in functional genomics. We have previously introduced a successful supervised pairwise ortholog classification approach implemented in a big data platform that considered several pairwise protein features and the low ortholog pair ratios found between two annotated proteomes (Galpert, D et al., *BioMed Research International*, 2015). The supervised models were built and tested using a *Saccharomycete* yeast benchmark dataset proposed by Salichos and Rokas (2011). Despite several pairwise protein features being combined in a supervised big data approach; they all, to some extent were alignment-based features and the proposed algorithms were evaluated on a unique test set. Here, we aim to evaluate the impact of alignment-free features on the performance of supervised models implemented in the Spark big data platform for pairwise ortholog detection in several related yeast proteomes.

**Results:**

The Spark Random Forest and Decision Trees with oversampling and undersampling techniques, and built with only alignment-based similarity measures or combined with several alignment-free pairwise protein features showed the highest classification performance for ortholog detection in three yeast proteome pairs. Although such supervised approaches outperformed traditional methods, there were no significant differences between the exclusive use of alignment-based similarity measures and their combination with alignment-free features, even within the twilight zone of the studied proteomes. Just when alignment-based and alignment-free features were combined in Spark Decision Trees with imbalance management, a higher success rate (98.71%) within the twilight zone could be achieved for a yeast proteome pair that underwent a whole genome duplication. The feature selection study showed that alignment-based features were top-ranked for the best classifiers while the runners-up were alignment-free features related to amino acid composition.

**Conclusions:**

The incorporation of alignment-free features in supervised big data models did not significantly improve ortholog detection in yeast proteomes regarding the classification qualities achieved with just alignment-based similarity measures. However, the similarity of their classification performance to that of traditional ortholog detection methods encourages the evaluation of other alignment-free protein pair descriptors in future research.

**Electronic supplementary material:**

The online version of this article (10.1186/s12859-018-2148-8) contains supplementary material, which is available to authorized users.

## Background

Homology between DNA or protein sequences is defined in terms of shared ancestry. Sequence regions that are homologous in species groups are referred to as conserved. Although useful as an aid in diagnosing homology, similarity is ill-suited as a defining criterion [1]. High sequence similarity might occur because of convergent evolution or the mere matching chance of non-related short sequences. Therefore such sequences are similar but not homologous [2]. Though sequence alignment is known as being the starting point in homology detection, this widely used method may also fail when the query sequence does not have significant similarities [3]. The mentioned pitfalls of homology detection based on sequence similarity are the grounds of the methods known as “alignment-free methods” [4, 5].

In homology regions, two segments of DNA may share ancestry because of either a speciation event (orthologs) or a duplication event (paralogs) [6]. The distinction between orthologs and paralogs is crucial since their concepts have different and important evolutionary and functional connotations. The combination of speciation and duplication events, along with horizontal gene transfers, gene losses, and genome rearrangements entangle orthologs and paralogs into complex webs of relationships. These semantics should be taken into account to clarify the descriptions of genome evolution [7].

Many graph-oriented [8–12], tree-oriented [13, 14] and hybrid-classified solutions [15–17] have arisen for ortholog detection. Graph-based algorithms are focused on pairwise genome comparisons by using similarity searches [18] to predict pairs or groups of ortholog genes (orthogroups) while tree-based ones follow phylogenetic criteria. In order to complement alignment-based sequence similarity, some approaches take into account conserved neighbourhoods in closely related species (synteny), genome rearrangements, evolutionary distances, or protein interactions [11, 15–17, 19–23]. Nevertheless, the effectiveness of such algorithms is still a challenge considering the complexity of gene relationships [24].

Some benchmark papers [25, 26] evaluate ortholog classification from functional or phylogenetic perspectives. However, ortholog genes are not always functionally similar [7] and single-gene phylogenies frequently yield erroneous results [27]. Consequently, and also due to the fact that contradictory results were found in a range of previous evaluation approaches, Salichos and Rokas proposed an evaluation scheme for ortholog detection using a benchmark *Saccharomycete* yeast dataset [27] built from Yeast Genome Order Browser (YGOB) database (version 1, 2005) [28]. The YGOB database includes yeast species that underwent a round of whole genome duplications and subsequent differential loss of gene duplicates; originating distinct gene retention patterns where in some cases the retained duplicates are paralogs. Such cases constitute “traps” for ortholog prediction algorithms. In detail, the YGOB database contains genomes of varying evolutionary distances, and the homology of several thousand of their genes has been accurately annotated through sequence similarity, phylogeny, and synteny conservation data. Hence, the evaluation scheme proposed by Salichos and Rokas implied the construction of a curated reference orthogroup dataset (“gold-groups”) deprived of paralogs to be compared with algorithm predictions on entire yeast proteomes. Actually, when extended versions of Reciprocal Best Hits (RBH) [29] and the Reciprocal Smallest Distance (RSD) [11] as well as Multiparanoid [30] and OrthoMCL [10] were evaluated using this benchmark dataset containing “traps”, they included paralogs in the orthogroups [27].

On the other hand, the massive growth of genomic data has required big data frameworks for high-performance processing of huge and varied data volumes [31]. Consequently, ortholog detection is an open bioinformatics field demanding either constant improvements in existing methods or new effective scaling algorithms to deal with big data. On the subject of big data [32], different platforms have been developed, such as Hadoop MapReduce [33], Apache Spark [34] and Flink [35] to implement classifiers.

In 2015, our group proposed a novel pairwise ortholog detection approach based on pairwise alignment-based feature combinations in a big data supervised classification scenario that manage the low ratio of ortholog pairs to non-ortholog pairs (millions of instances) in two yeast proteomes [36]. We built big data supervised models combining alignment-based similarity measures from global and local alignment scores, the length of sequences and the physicochemical profiles of proteins. We also proposed an evaluation scheme for supervised and unsupervised algorithms considering data imbalance. Big data supervised algorithms that manage data imbalance based on Random Forest outperformed three of the traditional unsupervised algorithms: Reciprocal Best Hits (RBH), Reciprocal Smallest Distance (RSD) and the Orthologous MAtrix (OMA). The latter was introduced quite recently and consists in an automated method and database for the inference of orthologs among entire genomes [12]. Despite the excellent results obtained with the supervised approach, the models were evaluated in a single pair of *Saccharomycete* yeast proteomes reported by Salichos et al. (2011)*.* In this paper, we intend to improve our previously reported big data supervised pairwise ortholog detection approach [36] as follows:

1. Evaluating the influence of alignment-free pairwise similarity measures on the classification performance of several supervised classifiers that consider data imbalance under the Spark platform [37].
2. Extending the test set to other related *Saccharomycete* yeast proteomes that constitute benchmark datasets with “traps” for ortholog detection algorithms.

Alignment-free similarity measures have shown several advantages over the alignment-based ones: (i) not sensitive to genome rearrangements, (ii) detection of functional signals at low sequence similarity and (iii) often less computationally complex and time consuming [4, 38]. In fact, they have been recently combined with alignment-based measures to fill some gaps in DNA and protein characterization left by these previous [39]. However, they have been poorly explored in ortholog detection algorithms; just *k*-mers counts were considered as a first step in the ortholog and co-ortholog assignment pipeline proposed by [38]. In this sense, several alignment-free protein features are used here to introduce pairwise similarity measures for ortholog detection across characterized yeast proteomes representing benchmark datasets. These alignment-free protein features are listed below, and most of them (5–10) are defined in the PROFEAT-Protein Feature Server [40] while four-color maps and Nandy’s descriptors (1–2) can be calculated by using our alignment-free graphical-numerical-encoding program [41] available at https://sourceforge.net/projects/ti2biop/. Generally, these protein features have been used to characterize functionally proteins at low sequence similarity using machine learning algorithms [42, 43].

1. Four color map descriptors: topological descriptors (spectral moments series) derived from protein four-colour maps [44].
2. Nandy’s descriptors: topological descriptors (spectral moments series) derived from Cartesian protein maps (Nandy’s DNA representation extended to proteins) [45].
3. *k*-mers or *k*-words: frequency of each subsequence or word of a fixed length *k* in a set of sequences [46].
4. Spaced *k*-mers or spaced words: contiguous *k*-mers with “don’t care characters” at fixed or pre-defined positions in a set of sequences [47].
5. Amino acid composition: the fraction of each amino acid within a protein [48, 49].
6. Chou’s pseudo amino acid composition descriptor: It is an improvement of the amino acid composition descriptor by adding information about the sequence order [50]. The sequence order is reached by the correlation between the most contiguous residues R*i*, R*j* placed at the topological distance *λ* from each other within the protein sequence. Further information can be found at http://www.csbio.sjtu.edu.cn/bioinf/PseAAC/type1.htm
7. Geary’s auto correlation**:** square autocorrelation of amino acid properties along the sequence [51].
8. Moran’s auto correlation: autocorrelation of amino acid properties along the sequence [52].
9. Total auto correlation: autocorrelation descriptors (Geary’s, Moran’s and Moreau-Broto’s) based on given amino acid properties are normalized all together [53].
10. Composition (C), Transition (T) and Distribution (D) (CTD) descriptors: information from the division of amino acid into three classes according to the value of its attributes e.g. hydrophobicity, normalized van der Waals volume, polarity, etc. So, each amino acid is classified by each one of the indices into class 1, 2 and 3. C descriptors: the global percent for each encoded class (1, 2 and 3) in the sequence, T descriptors: the percentage frequency to which class 1 is followed by class 2 or 2 is followed by 1 in the encoded sequence. D descriptors: the distribution of each attribute in the encoded sequence [54, 55].
11. Quasi-Sequence-Order (QSO) descriptors: combination of sequence composition and correlation of amino acid properties defined by Chou KC (2000) [56].

In order to evaluate the influence of the alignment-free features on ortholog detection, we build three kinds of supervised pairwise ortholog detection models (i) one based on previously reported alignment-based pairwise protein features (global and local alignment scores and the physicochemical profiles) (ii) a new one incorporating only the alignment-free features listed above and (iii) another one resulting from the combination of alignment-based and alignment-free protein features. For model building we are using different machine learning algorithms (Random Forest, Decision Trees, Support Vector Machines, Logistic Regression and Naïve Bayes) implemented in the Spark big data architecture as well the gold-groups reported by Salichos and Rokas in 2011. Each supervised approach was evaluated in several benchmark yeast proteome pairs containing “traps” for ortholog detection [27]. The evaluation scheme allows the performance comparison of the supervised pairwise ortholog detection algorithms against RBH, RSD and OMA considering the imbalance between orthologs/non-orthologs in yeast proteomes, as can be seen in our previous work [36]. Moreover, a feature selection study is carried out to evaluate the importance of the new alignment-free similarity measures and the previously reported alignment-based as well as the alignment-based + alignment-free features combination over the ortholog detection.

Spark classifiers are introduced here since they manage complete datasets instead of the ensemble of classifiers built with the corresponding data in partitions as in Hadoop MapReduce implementations. The Spark random oversampling may also speed up the pre-processing while the resampling size parameter value over 100% may improve the classification of the minority class in extremely high imbalanced datasets [57] like pairwise proteome comparison ones. All these improvements in the algorithm architecture together with the inclusion of alignment-free features may have a positive effect on the classification quality and the speed of convergence.

As a result of the experiments in this study, the advantages of the Spark big data architecture over MapReduce implementations in terms of classification performance and execution time for supervised pairwise ortholog detection have been confirmed, conversely, the introduction of alignment-free features into several supervised classifiers that use alignment-based similarity measures did not significantly improve the pairwise ortholog detection. In fact, the feature selection study showed that alignment-based similarity measures are more relevant for the supervised ortholog detection than alignment-free features. However, many of the supervised big data classifiers built with both alignment-based and alignment-free features surpassed the traditional methods like RBH, RSD and OMA in three pairs of yeast proteomes. Precisely, some of these tree-based supervised classifiers could detect more ortholog pairs at the twilight zone (< 30% of protein identity) in two whole-duplicated genomes. These findings encourage us to keep on working on improving our alignment-free protein features in order to fill the gap of the alignment algorithms when genetic events blur the ortholog detection.

## Methods

### Alignment-based similarity measures

We have previously defined the following alignment-based similarity measures for protein pairs found in two yeast proteomes *P*_1_ = {*x*_1_, *x*_2_, …, *x*_*n*_} and *P*_2_ = {*y*_1_, *y*_2_, …, *y*_*m*_} in [36]:

- *S*_1_: Similarity based on global alignment scores.
- *S*_2_: Similarity based on local alignment scores.
- *S*_3_: Similarity based on the physicochemical profile from matching regions (with no gaps) of aligned sequences at different window sizes (*W* = 3, 5 and 7).

and *S*_4_: Similarity based on the pairwise differences of protein lengths. Despite *S*_4_ being included with the previous (*S*_1_…*S*_3_), it is not an alignment-dependent measure. All these similarity measures were normalized by the maximum value.

### Alignment-free similarity measures

Protein sequences from yeast proteomes are turned into numerical vectors using the alignment-free methods listed in the background section. The Pearson correlation coefficient was selected as an alignment-free similarity measure between two numerical vectors. The selection is based on the valuable information obtained with the significance value of the Pearson coefficient [58]. Each alignment-free pairwise similarity is calculated as follows:

1 $$ {S}_k\left({x}_i,{y}_j\right)=\left\{\begin{array}{cc} Corr\left( AAX, AAY\right)&, sig\le 0.05\\ {}0&, sig>0.05\end{array}\right.,k=\mathrm{5..26} $$

where *AAX* and *AAY* represent the numerical vectors of proteins *x*_*i*_ and *y*_*j*_, respectively.

The alignment-free pairwise similarity measures evaluated in this study (*S*_5_-*S*_26_) are listed below. Each pairwise similarity measure is labelled by its corresponding alignment-free method and the main parameters used.

- *S*_5_: Similarity based on amino acid composition.
- *S*_6_: Similarity based on pseudo-amino acid composition with *λ* = 4. The parameter *λ* is the topological distance between two amino acids in the sequence pseudo-amino acid composition concept where the sequence order effect is integrated to the amino acid composition, *λ* < protein length.
- *S*_7_: Similarity based on pseudo amino acid composition with *λ* = 3.
- *S*_8_: Similarity based on pseudo amino acid composition with *λ* = 10.
- *S*_9_: Similarity based on *k*-mers composition with *k* = 3 where *k* represents the size of contiguous words (matching positions).
- *S*_10_: Similarity based on *k*-mers composition with *k* = 2.
- *S*_11_: Similarity based on Geary’s auto correlation.
- *S*_12_: Similarity based on Moran’s auto correlation.
- *S*_13_: Similarity based on Total auto correlation.
- *S*_14_: Similarity based on Composition, Distribution and Transition (Composition).
- *S*_15_: Similarity based on Composition, Distribution and Transition (Distributions).
- *S*_16_: Similarity based on Composition, Distribution and Transition (Transition).
- *S*_17_: Similarity based on Composition, Distribution and Transition (Total).
- *S*_18_: Similarity based on four-color maps.
- *S*_19_: Similarity based on spaced *k*-mers/spaced words composition with *k* = 2 (matching positions (1)) and one “don’t care positions” (0); patterns: “101”.
- *S*_20_: Similarity based on *k*-mers/spaced words composition with *k* = 2 and two “don’t care positions”; patterns: “1001”.
- *S*_21_: Similarity based on spaced *k*-mers/spaced words composition with *k* = 2 and three “don’t care positions”; patterns: “10,001”.
- *S*_22_: Similarity based on spaced *k*-mers/spaced words composition with *k* = 3 and one “don’t care positions”; patterns: “1101”, “1011”.
- *S*_23_: Similarity based on spaced *k*-mers/spaced words composition with *k* = 3 and two “don’t care positions”; patterns: patterns: “10,011”, “10,101”, “11,001”.
- *S*_24_: Similarity based on spaced *k*-mers/spaced words composition with *k* = 3 and three “don’t care positions”; patterns: “100,011”, “110,001”, “101,001”, “100,101”.
- *S*_25_: Similarity based on Nandy’s descriptor.
- *S*_26_: Similarity based on Quasi-Sequence-Order with maxlag = 30.

As the same measure or function (Pearson correlation) is used to quantify the previously-mentioned alignment-free pairwise similarities; thus, we are definitely evaluating the corresponding alignment-free protein features giving rise to them.

### Pairwise ortholog detection based on big data supervised models managing ortholog/non-ortholog imbalance

**Fig. 1:**
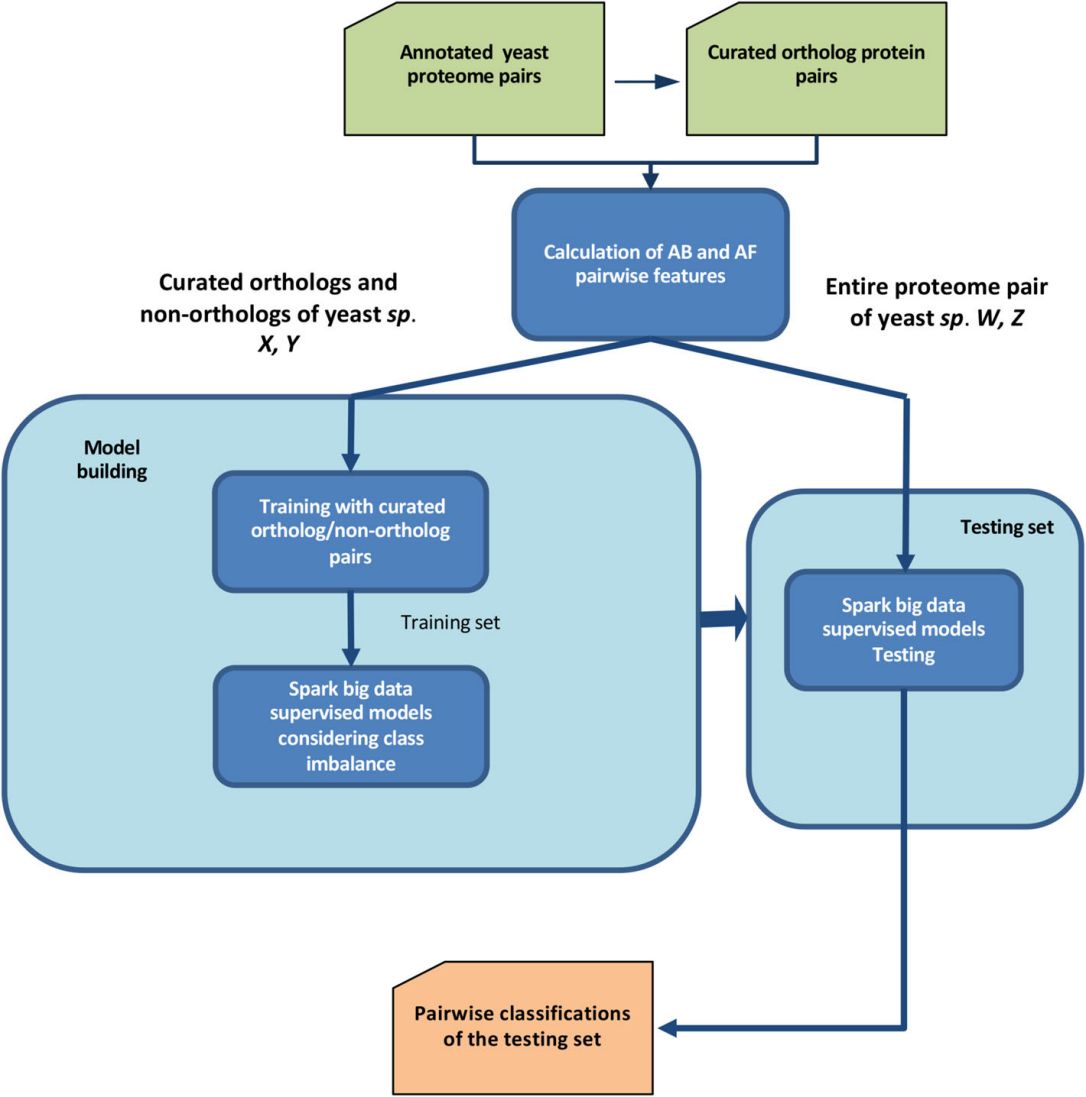
Flowchart of Spark imbalanced big data classification pairwise ortholog detection algorithms

The general classification scheme for pairwise ortholog detection using supervised big data algorithms managing the ortholog/non-ortholog imbalance found in yeast proteome pairs is represented in Fig. 1. First, pairwise similarity (alignment-based and alignment-free) measures are calculated for all annotated proteome pairs. Secondly, pairwise curated classifications (ortholog and non-ortholog pairs) should be extracted from ortholog curated datasets or gold-groups [27] with the aim of training/building the prediction models. The new Spark big data supervised models are based on Random Forest, Decision Trees, Support Vector Machines, Logistic Regression or Naive Bayes algorithms (Tables 1, 1–5). The other model (6 in Table 1) represents the Random Forest version implemented in Hadoop MapReduce. Thus, the big data pairwise ortholog detection models are built with curated classifications from any proteome pair of the “gold-groups” and tested on entire proteome pairs (not included in training) containing paralogs. In this way, built models can be generalized to multiple genome/proteome pairs since the model building step can be executed once.

**Table 1:** Big data supervised algorithms, imbalance management pre-processing methods and parameter values considered in this paper

N	Algorithms	Pre-processing	Parameter values	
1	Spark Random Forest^a^	ROS/RUS	*NumTrees*: 100 (by default) *MaxBins*: 1000 (by default) *Impurity*: gini/entropy	*MaxDepth*: 5 (by default) *Number of maps*: 20 *MinInstancesPerNode*: 2 *MinInfoGain*: 0 *FeatureSubsetStrategy*: auto *Resampling size*: 100%/130%
2	Spark Decision Trees^b^	ROS/RUS	*MaxBins* - > Number of bins used when discretizing continuous features: 100 (by default) *Impurity* - > Impurity measure: gini (by default) *MaxDepth* - > Maximum depth of each tree: 5 (by default) *MinInstancesPerNode*: 2 *MinInfoGain*: 0 *FeatureSubsetStrategy*: auto *Resampling size*: 100%/130%	
3	Spark Support Vector Machines^c^	ROS	*Regulation parameter*: 1.0/0.5/0.0 *Number of iterations*: 100 (by default)	*StepSize*: 1.0 (by default) *miniBatchFraction*: 1.0 *Resampling size*: 100%/130%
4	Spark Logistic Regression^d^	ROS	*Number of iterations*: 100 (by default) *StepSize* - > Stochastic gradient descent parameter: 1.0 (by default)	*MiniBatchFraction* - > Fraction of the dataset sampled and used in each iteration: 1.0 (by default: 100%) *Resamplig size*: 100%/130%
5	Spark Naive Bayes^e^	ROS	*Additive smoothing Lambda*: 1.0 (by default)	*Resampling size*: 100%/130%
6	MapReduce Random Forests^f^	ROS	*Number of trees*: 100 *Random selected attributes per node*: 3	*Number of maps*: 20 *Resampling size*: 100%/130%

ROS: Random Oversampling, RUS: Random Undersampling

^a^ https://spark.apache.org/docs/latest/mllib-ensembles.html

^b^ https://spark.apache.org/docs/latest/mllib-decision-tree.html

^c^ https://spark.apache.org/docs/latest/mllib-linear-methods.html#linear-support-vector-machines-svms

^d^ https://spark.apache.org/docs/latest/mllib-linear-methods.html#logistic-regression

^e^ https://spark.apache.org/docs/latest/mllib-naive-bayes.html

^f^Random Forest implementation available in https://mahout.apache.org/

The training step involves the ortholog/non-ortholog imbalance management, and the testing step includes the selection of the adequate quality measures for imbalance datasets. The main pre-processing algorithms proposed to cope with data imbalance are labelled as ROS (Random Oversampling) and RUS (Random Undersampling). The Spark implementation of these algorithms are available at a spark-packages site https://spark-packages.org/package/saradelrio/Imb-sampling-ROS_and_RUS [59]. The new proposed Spark big data classifiers with their parameter values (Table 1) are implemented in the Spark MLlib Machine Learning library [60].

The performance of the big data supervised models shown in Table 1 is compared with unsupervised reference algorithms like Reciprocal Best Hits (RBH), Reciprocal Smallest Distance (RSD) and Orthologous MAtrix (OMA) following the evaluation scheme described below. These unsupervised algorithms are specified in Table 2 with their parameter values.

**Table 2:** Unsupervised reference algorithms and parameter values proposed in [36]

Algorithms	Parameter values
Reciprocal Best Hits (RBH)^a^	*Filter*: soft *Alignment*: Smith Waterman *E-value*: 1e-06
Reciprocal Smallest Distance (RSD)^b^	*E-value thresholds*: 1e-05, 1e-10 and 1e-20 *Divergence thresholds* α: 0.8, 0.5 and 0.2
Orthologous MAtrix (OMA)^c^	Default parameter values

^a^Matlab script and BLAST program available in http://www.ncbi.nlm.nih.gov/BLAST/

^b^Phyton script available in https://pypi.python.org/pypi/reciprocal_smallest_distance/1.1.4/

^c^Stand-alone version available in http://omabrowser.org/standalone/OMA.0.99z.3.tgz

### Evaluation scheme

In order to evaluate the performance of pairwise ortholog detection algorithms we use the gold-groups (deprived of paralogs) retrieved by Salichos and Rokas [27] from the YGOB database (version 1, 2005) [28]. Such gold-groups are split into two subgroups. The first one contains all orthologs from species not subjected to a whole genome duplication (pre-WGD) together with all orthologs from species that underwent a whole genome duplication (post-WGD) resulting in two chromosome segments (track A and B) found on track A, whereas the second subgroup contains the same orthologous genes from pre-WGD species together with all orthologs from post-WGD species found on track B.

The evaluation scheme includes the following steps:

1. Data splitting into two training and testing sets. The training process is carried out by using curated ortholog pairs (positive set) found either in pre-WGD species or in track A/B of post-WGD species. Similarly, a curated negative set is made up of all possible non-ortholog pairs found between two yeast proteomes deprived of paralogs (gold-groups).
2. The testing step is carried out on entire proteome pairs excluding the pairs used in learning steps. Test sets are made up of all possible annotated protein pairs (orthologs, non-orthologs and paralogs) found between pre-pre WGD or pre-post WGD or post-post WGD yeast species pairs. Three of the traditional unsupervised algorithms (RBH, RSD and OMA) for pairwise ortholog detection were also comparatively evaluated on the test sets.
3. The performance evaluation of both methods (supervised vs. unsupervised ortholog detection) is based on previously curated classifications; so, curated orthologs and non-orthologs are considered as “true positives” (*TP*) and “true negatives” (*TN*), respectively. Paralogs are considered as “traps” for ortholog detection algorithms because they can be easily misclassified as “orthologs”. The selected evaluation metrics *AUC*, *G-Mean, TP*_*Rate*_ (*TPR*) and *TN*_*Rate*_ (*TNR*) are suitable for imbalanced datasets [36].

### Datasets

Annotated proteome pairs from related yeast species of the *Saccharomycete* yeast class (pre-WGD *Kluyveromcyes lactis* and *Kluyveromyces waltii* and post-WGD *Saccharomyces cerevisiae* and *Candida glabrata*) are selected in order to analyze the quality of our approach. Table 3 shows the details of the proteome pairs (*S. cerevisiae - K. lactis*, *S. cerevisiae* - *C. glabrata*, *C. glabrata* - *K. lactis*, and *K. lactis - K. waltii*). We include the total number of pairwise features, the total of protein pairs per class and the imbalance ratio (*IR*).

**Table 3:** Datasets used in the experiments

Dataset id	Proteome pair	Number of protein features	Protein pair per class (non-orthologs; orthologs)	Imbalance ratio *(IR)*
*ScerKlac*	*S. cerevisiae - K. lactis*	29	(31,218,485; 3062)	10,195.456
*ScerCgla*	*S. cerevisiae - C. glabrata*	29	(30,562,272; 2843)	10,750.008
*CglaKlac*	*C. glabrata - K. lactis*	29	(27,778,732; 1573)	17,659.715
*KlacKwal*	*K. lactis - K. waltii*	29	(27,772,372; 2606)	10,657.088

Protein sequences of the previously listed proteomes can be found in Additional file 1.

### Experiments

Three study cases were designed to inspect the influence of the alignment-free features on the supervised classification for ortholog detection. Thus, big data supervised classifiers are compared considering three study cases: alignment-based features, alignment-free features and alignment-based + alignment-free features. Specifically, in the alignment-based case we use similarity measures *S*_1..3_ with *S*_3_ calculated by using windows sizes 3, 5 and 7. In the alignment-free case we use *S*_4..27_ and then, in the alignment-based + alignment-free case we use all the similarity measures. The different models to be compared are built with *ScerKlac* and tested in *ScerCgla, CglaKlac* and *KlacKwal* datasets*.* The algorithms in Table 1 and Table 2 were executed in two experiments: (i) Algorithm Performance Experiment and (ii) Feature Importance Experiment. In the experiment (i), the classification performance of supervised algorithms in the three study cases was contrasted with the one achieved by the traditional ortholog detection methods: RBH, RSD and OMA. Additionally, the identification of orthologs at the twilight zone (remote orthologs) was also included in this experiment as well as the execution time of the most Spark predictive algorithms was also collected together with Hadoop MapReduce Mahout implementations for comparative purposes. Then, in experiment (ii), the importance of both alignment-based and alignment-free features and their combinations was also studied in ortholog classification. The MLlib version used in experiment (i) is 1.6 while in experiment (ii) the 2.0 version allows the Random Forest model exploration to determine the feature importance.

## Results

### Algorithm performance

The classification quality measures *G-Mean* and *AUC* for Decision Trees, Random Forest, Logistic Regression, Naive Bayes and Support Vector Machines for the study cases with alignment-based, alignment-free and alignment-based + alignment-free features are shown in Table 4. The same measures for RBH, RSD and OMA are also included in this table. The underlined values highlight the most effective methods in this experiment while the bold values identify the best performing supervised and unsupervised algorithms in each testing dataset. The best *AUC* and *G-Mean* values (0.9977) correspond to the ROS (130% resampling) and RUS pre-processed Spark Random Forests in the *ScerCgla* and *KlacKwal* datasets with the alignment-based features as well as to the ROS (100% resampling) Spark Decision Trees in the *ScerCgla* dataset with the alignment-based + alignment-free feature combinations. These *G-Mean* results outperformed the best value of 0.9941 reported in our previous paper [36] for *ScerCgla* with a version of Hadoop MapReduce Random Forest**.** The best values (*AUC* = 0.9486) of the unsupervised classifiers correspond to RSD 0.8 1e-05 (α = 0.8 and E-value = 1e-05 recommended in [61]). This traditional ortholog detection method outperformed most of the supervised algorithms built with alignment-free features except when ROS (100% resampling) was applied to Spark Decision Trees in *ScerCgla* (*AUC* = 0.9496).

**Table 4:** The *AUC* and *G-Mean* values of all the algorithms (supervised and unsupervised) in testing datasets

Algorithm/Dataset	Alignment-based Features						Alignment-free Features						Alignment-based + Alignment-free Features					
	*G-Mean*			*AUC*			*G-Mean*			*AUC*			*G-Mean*			*AUC*		
	*Scer* *Cgla*	*Cgla* *Klac*	*Klac* *Kwal*	*Scer* *Cgla*	*Cgla* *Klac*	*Klac* *Kwal*	*Scer* *Cgla*	*Cgla* *Klac*	*Klac* *Kwal*	*Scer* *Cgla*	*Cgla* *Klac*	*Klac* *Kwal*	*Scer* *Cgla*	*Cgla* *Klac*	*Klac* *Kwal*	*Scer* *Cgla*	*Cgla* *Klac*	*Klac* *Kwal*
Supervised Algorithms																		
Spark Random Forest MLlib 1.6 (*Impurity*: gini)																		
Normal	0.3853	0.3119	0.3421	0.5742	0.5486	0.5585	0.0000	0.0000	0.0000	0.5000	0.5000	0.5000	0.6647	0.1009	0.6104	0.7209	0.5051	0.6863
ROS-100	0.9962	0.9941	0.9966	0.9962	0.9941	0.9966	0.9375	0.9139	0.9186	0.9375	0.9148	0.9189	0.9972	0.9917	0.9950	0.9972	0.9917	0.9950
ROS-130	**0.9977**	**0.9956**	0.9974	**0.9977**	**0.9956**	0.9974	0.9313	0.9162	0.9166	0.9315	0.9166	0.9166	0.9958	0.9929	0.9945	0.9958	0.9930	0.9945
RUS	0.9974	0.9953	**0.9977**	0.9974	0.9953	**0.9977**	0.9325	0.8917	0.9152	0.9325	0.8941	0.9153	0.9973	0.9950	0.9973	0.9973	0.9950	0.9973
Spark Random Forest MLlib 1.6 (*Impurity*: entropy)																		
Normal	0.7457	0.0365	0.3809	0.7780	0.1192	0.5725	0.0000	0.0000	0.0000	0.5000	0.0858	0.5000	0.6001	0.0064	0.3195	0.6801	0.0064	0.5510
ROS-100	0.9971	0.9948	0.9969	0.9971	0.9948	0.9969	0.9333	**0.9169**	0.9097	0.9333	**0.9180**	0.9106	0.9971	0.9947	0.9965	0.9971	0.9947	0.9965
ROS-130	0.9974	0.9950	0.9967	0.9974	0.9950	0.9967	0.9267	0.9101	0.9087	0.9267	0.9108	0.9088	0.9975	**0.9955**	0.9945	0.9975	**0.9955**	0.9945
RUS	**0.9977**	0.9949	0.9976	**0.9977**	0.9949	0.9976	0.9396	0.9081	0.9202	0.9397	0.9097	0.9207	0.9974	0.9948	**0.9975**	0.9974	0.9948	**0.9975**
Spark Decision Trees MLlib 1.6																		
Normal	0.3751	0.2983	0.3301	0.5703	0.5445	0.5545	0.3848	0.0252	0.3548	0.5740	0.5003	0.5629	0.6505	0.5017	0.6107	0.7115	0.6259	0.6865
ROS-100	0.9973	0.9941	0.9960	0.9973	0.9941	0.9960	**0.9496**	0.9153	0.9258	**0.9496**	0.9157	0.9262	**0.9977**	0.9483	0.9954	**0.9977**	0.9495	0.9954
ROS-130	0.9957	0.9906	0.9961	0.9957	0.9906	0.9961	0.9464	0.8993	0.9293	0.9465	0.9002	0.9293	0.9972	0.9449	0.9965	0.9972	0.9463	0.9965
RUS	0.9970	0.9936	0.9975	0.9970	0.9936	0.9975	0.9473	0.9156	**0.9317**	0.9473	0.9158	**0.9317**	0.9971	0.9720	0.9966	0.9971	0.9723	0.9966
Spark Support Vector Machines MLlib 1.6																		
Normal (0.0)	0.0000	0.0000	0.0000	0.5000	0.5000	0.5000	0.0000	0.0000	0.0000	0.5000	0.5000	0.5000	0.0000	0.0000	0.0000	0.5000	0.5000	0.5000
Normal (0.5)	0.0000	0.0000	0.0000	0.5000	0.5000	0.5000	0.0000	0.0000	0.0000	0.5000	0.5000	0.5000	0.0000	0.0000	0.0000	0.5000	0.5000	0.5000
Normal (1.0)	0.0000	0.0000	0.0000	0.5000	0.5000	0.5000	0.0000	0.0000	0.0000	0.5000	0.5000	0.5000	0.0000	0.0000	0.0000	0.5000	0.5000	0.5000
ROS-100 (0.0)	0.0000	0.0000	0.0000	0.5000	0.5000	0.5000	0.8486	0.8467	0.8482	0.8517	0.8482	0.8496	0.9682	0.9581	0.9677	0.9684	0.9585	0.9679
ROS-100 (0.5)	0.0000	0.0000	0.0000	0.5000	0.5000	0.5000	0.0000	0.0000	0.0000	0.5000	0.5000	0.5000	0.0000	0.0000	0.0000	0.5000	0.5000	0.5000
ROS-100 (1.0)	0.0000	0.0000	0.0000	0.5000	0.5000	0.5000	0.0000	0.0000	0.0000	0.5000	0.5000	0.5000	0.0000	0.0000	0.0000	0.5000	0.5000	0.5000
ROS-130 (0.0)	0.0000	0.0000	0.0000	0.5000	0.5000	0.5000	0.7719	0.7786	0.7779	0.7929	0.7950	0.7961	0.9708	0.9612	0.9683	0.9709	0.9615	0.9685
ROS-130 (0.5)	0.0000	0.0000	0.0000	0.5000	0.5000	0.5000	0.0000	0.0000	0.0000	0.5000	0.5000	0.5000	0.0000	0.0000	0.0000	0.5000	0.5000	0.5000
ROS-130 (1.0)	0.0000	0.0000	0.0000	0.5000	0.5000	0.5000	0.0000	0.0000	0.0000	0.5000	0.5000	0.5000	0.0000	0.0000	0.0000	0.5000	0.5000	0.5000
Spark Logistic Regression MLlib 1.6																		
Normal	0.0000	0.0000	0.0000	0.5000	0.5000	0.5000	0.0000	0.0000	0.0000	0.5000	0.5000	0.5000	0.0000	0.0000	0.0000	0.5000	0.5000	0.5000
ROS-100	0.3994	0.3663	0.3943	0.5012	0.4848	0.4981	0.2861	0.2867	0.2725	0.5028	0.5032	0.4989	0.0815	0.0665	0.0677	0.5007	0.4995	0.4996
ROS-130	0.4056	0.3925	0.4060	0.5006	0.5089	0.5003	0.3008	0.3091	0.2954	0.5027	0.5054	0.5012	0.1416	0.1173	0.1274	0.5018	0.4987	0.4999
Spark Naive Bayes MLlib 1.6																		
Normal	0.0000	0.0000	0.0000	0.5000	0.5000	0.5000	0.0000	0.0000	0.0000	0.5000	0.5000	0.5000	0.0000	0.0000	0.0000	0.5000	0.5000	0.5000
ROS-100	0.4070	0.3943	0.4002	0.4990	0.4981	0.4949	0.4182	0.4371	0.4164	0.5009	0.5113	0.4999	0.1365	0.1498	0.1180	0.4996	0.5016	0.4972
ROS-130	0.0171	0.4060	0.0172	0.5001	0.5003	0.5001	0.4823	0.4991	0.4825	0.4997	0.5202	0.4985	0.2067	0.2163	0.1953	0.5003	0.5024	0.4979
MapReduce Random Forest Mahout 0.9																		
Normal	0.7178	0.6652	0.6864	0.7576	0.7212	0.7356												
ROS-100	0.9903	0.9786	0.9859	0.9903	0.9789	0.9860												
ROS-130	0.9905	0.9783	0.9846	0.9905	0.9785	0.9847												
Unsupervised Algorithms																		
RBH	0.8069	0.8052	0.8491	0.8255	0.8242	0.8605												
RSD 0.2 1e-20	0.9309	0.9038	0.9654	0.9333	0.9092	0.966												
RSD 0.5 1e-10	0.9426	0.9277	0.9818	0.9442	0.9294	0.9819												
RSD 0.8 1e-05	**0.9472**	**0.9373**	**0.9876**	**0.9486**	**0.9374**	**0.9877**												
OMA	0.7311	0.7264	0.9388	0.7673	0.9163	0.9407												

Supervised algorithm performance is presented for the alignment-based, alignment-free and alignment-based + alignment-free feature combinations. The best results in each dataset are in bold face and the general best results are underlined. The Random Oversampling pre-processing (ROS) is accompanied by the corresponding resampling size value. RSD parameter values are the divergence and the *E-value* thresholds. Support Vector Machines are represented with their regulation parameter values

Table 5 shows the percent of true positives obtained by the outstanding supervised classifiers and the reference methods in the identification of curated orthologs pairs found at the twilight zone among the studied yeast proteome pairs. The corresponding percent of true positives for the study cases with alignment-based, alignment-free and alignment-based + alignment-free features are also included for the selected supervised classifiers. The underlined value represents the most effective method while the bold values identify the best performing algorithms in each testing dataset.

**Table 5:** Percentage of true positives (%TP) identified by both outstanding supervised and unsupervised classifiers when detecting ortholog pairs in the twilight zone (< 30% of identity)

Algorithm/Dataset	Alignment-based Features			Alignment-free Features			Alignment-based + Alignment-free Features		
	%TP			%TP			%TP		
Supervised Algorithms	*Scer* *Cgla*	*Cgla* *Klac*	*Klac* *Kwal*	*Scer* *Cgla*	*Cgla* *Klac*	*Klac* *Kwal*	*Scer* *Cgla*	*Cgla* *Klac*	*Klac* *Kwal*
Spark Random Forest MLlib 1.6									
Normal	0.00	0.00	0.00	0.00	0.00	0.00	3.54	0.00	2.25
ROS-100	97.43	96.26	98.03	71.06	64.29	57.87	96.14	91.84	93.54
ROS-130	98.71	**96.94**	98.31	76.21	64.97	65.45	95.18	93.88	93.54
RUS	**99.04**	96.26	98.60	74.60	64.29	61.24	96.78	93.88	95.51
Spark Decision Trees MLlib 1.6									
Normal	0.32	0.68	0.28	0.00	0.00	0.56	12.54	7.82	9.55
ROS-100	95.18	94.56	97.19	72.67	62.93	55.62	97.75	84.69	96.07
ROS-130	95.82	91.50	97.47	79.74	61.56	63.48	98.71	87.41	96.35
RUS	98.07	95.24	**99.16**	76.53	67.01	65.45	98.07	90.82	97.47
Unsupervised Algorithms									
RBH	57.56	58.84	73.31						
RSD 0.2 1e-20	46.95	45.92	62.36						
RSD 0.5 1e-10	61.41	61.90	80.34						
RSD 0.8 1e-05	68.17	70.41	85.96						
OMA	42.77	45.24	46.91						

The best results in each dataset are in bold face and the general best results are underlined. Supervised algorithm performance is presented for the alignment-based, alignment-free and alignment-based + alignment-free feature combinations

The ortholog pairs placed in the twilight zone are: 311 out of 30,558,738 *ScerCgla* protein pairs, 294 out of 27,775,380 *CglaKlac* pairs and 356 out of 27,770,047 *KlacKwal* pairs. The highest true positive percentage (99.16%) corresponded to the RUS pre-processed Spark Decision Trees in the *KlacKwal* dataset with alignment-based features. On the other datasets, the best true positive percentages were also obtained with the alignment-based features; 99.04% and 96.94% that corresponded to the RUS pre-processed Spark Random Forest in *ScerCgla*, and to the ROS (130% resampling) Spark Random Forest in *CglaKlac*, respectively. In total, alignment-based features by themselves and alignment-based + alignment-free feature combinations surpassed the alignment-free and the classical unsupervised approaches. Generally, the alignment-free feature-based classifiers with imbalance management outperformed the unsupervised classifiers in each dataset, with the exceptions of the best RSD classifiers (RSD 0.8 1e-05) and (RSD 0.5 1e-10) in *CglaKlac* and *KlacKwal*, and the RBH classifier in *KlacKwal*. The Spark Decision Trees improved their performance with the combination of alignment-based and alignment-free features in *ScerCgla*, two yeast species that underwent a single round of whole genome duplications with subsequent gene losses. Specifically, the ROS (130% resampling) Decision Trees equalled the second best result (98.71%) of the ROS (130% resampling) Spark Random Forest in such a complex dataset.

Run time is presented in Table 6 for Random Forest Spark and Hadoop MapReduce variants as well as for Spark Decision Trees. Some of the highlighted time values of Spark Random Forest with RUS correspond to its best quality performance values obtained with alignment-based features. At the same time, some of the quickest underlined ROS (100% resampling) time values of Decision Trees coincide with the best quality results in the highest dimension alignment-based + alignment-free case. Differences in time between Spark and Hadoop MapReduce Random Forest are noticeable while classification quality values are improved for the evaluated Spark version.

**Table 6:** Run time values (hh:mm:ss) comprising learning and classifying steps obtained by the highest quality Spark supervised algorithms (Decision Trees and Random Forest) together with the corresponding values of the Hadoop MapReduce Random Forest implementation. Supervised algorithm run time values are presented for the alignment-based, alignment-free and alignment-based + alignment-free feature combinations. The Random Oversampling pre-processing (ROS) is accompanied by the corresponding resampling size value

Algorithm/Dataset	Alignment-based Features			Alignment-free Features			Alignment-based + Alignment-free Features		
	*ScerCgla*	*CglaKlac*	*KlacKwal*	*ScerCgla*	*CglaKlac*	*KlacKwal*	*ScerCgla*	*CglaKlac*	*KlacKwal*
Spark Random Forest MLlib 1.6									
NORMAL Learn	00:00:49	00:00:57	00:00:57	00:01:03	00:01:05	00:01:07	00:00:57	00:00:57	00:01:00
NORMAL Classify	00:00:19	00:00:38	00:00:24	00:00:31	00:00:26	00:00:25	00:00:34	00:00:30	00:00:32
ROS-100 Learn	00:01:43	00:02:34	00:02:29	00:01:48	00:01:43	00:01:47	00:01:48	00:01:50	00:01:48
ROS-100 Classify	00:00:20	00:00:19	00:00:19	00:00:33	00:00:28	00:00:29	00:00:33	00:00:31	00:00:31
ROS-130 Learn	00:02:09	00:02:15	00:02:43	00:02:03	00:01:57	00:02:00	00:02:06	00:02:03	00:01:57
ROS-130 Classify	00:00:19	00:00:18	00:00:18	00:00:39	00:00:30	00:00:34	00:00:41	00:00:31	00:00:34
RUS Learn	**00:00:09**	**00:00:09**	**00:00:09**	**00:00:11**	**00:00:11**	**00:00:11**	**00:00:10**	**00:00:10**	**00:00:10**
RUS Classify	**00:00:14**	**00:00:14**	**00:00:13**	**00:00:39**	**00:00:31**	**00:00:42**	**00:00:41**	**00:00:39**	**00:00:39**
Spark Decision Trees MLlib 1.6									
NORMAL Learn	00:00:31	00:00:31	00:00:35	00:00:35	00:00:33	00:00:35	00:00:49	00:00:38	00:00:40
NORMAL Classify	00:00:13	00:00:12	00:00:15	00:00:23	00:00:20	00:00:20	00:00:25	00:00:25	00:00:24
ROS-100 Learn	00:00:57	00:00:58	00:00:56	00:00:59	00:01:03	00:01:01	00:01:11	00:01:07	00:01:07
ROS-100 Classify	00:00:11	00:00:15	00:00:13	00:00:22	00:00:20	00:00:24	00:00:24	00:00:23	00:00:24
ROS-130 Learn	00:00:57	00:00:58	00:00:57	00:01:14	00:01:06	00:01:05	00:01:15	00:01:13	00:01:16
ROS-130 Classify	00:00:12	00:00:19	00:00:11	00:00:23	00:00:22	00:00:22	00:00:25	00:00:24	00:00:23
RUS Learn	**00:00:08**	**00:00:08**	**00:00:08**	**00:00:09**	**00:00:09**	**00:00:09**	**00:00:09**	**00:00:09**	**00:00:09**
RUS Classify	**00:00:12**	**00:00:11**	**00:00:11**	**00:00:33**	**00:00:26**	**00:00:34**	**00:00:36**	**00:00:34**	**00:00:35**
MapReduce Random Forest Mahout 0.9									
NORMAL Learn	23:25:10	23:25:10	23:25:10						
NORMAL Classify	00:14:25	00:13:07	00:13:04						

### Feature importance

The feature importance study carried out in the *ScerCgla* dataset is summarized in Table 7 for the three feature cases (alignment-based, alignment-free and alignment-based + alignment-free). The entropy value of each feature in the Spark tree-based models obtained after RUS pre-processing was calculated with the Weka software [62] in addition to the average impurity decrease. The number of nodes that included certain features in the Random Forest building with RUS pre-processing was also estimated. The decrease of the average impurity for the Random Forest with ROS variants implemented in the MLlib 2.0 library was incorporated in this table too. Bold values represent high-importance features while underlined values emphasize the best values.

**Table 7:** Feature importance calculated for the highest quality Spark supervised algorithms (Decision Trees (DT) and Random Forest (RF)). The entropy, the number of nodes that included certain features in the Random Forest building with RUS pre-processing and the average impurity decrease of the MLlib 2.0 Random Forest with ROS variants are presented for the alignment-based, alignment-free and alignment-based + alignment-free feature combinations The Random Oversampling pre-processing (ROS) is accompanied by the corresponding resampling size value

	RUS + DT-Spark Weka	RUS + RF-Spark/Gini Weka		RF MLlib 2.0-Spark/Gini (Avg. Impurity Decrease)			
	Entropy	Avg. Impurity Decrease	Number of Nodes	Normal	ROS-100	ROS-130	RUS
Alignment-based Features/Algorithm							
*nw*	**0.789**	**0.520**	42	**0.809**	0.180	0.175	0.171
*sw*	**0.982**	**0.360**	**802**	0.035	**0.642**	**0.647**	**0.647**
*profile3*	**0.783**	**0.360**	**417**	0.043	0.167	0.167	0.167
*profile5*	0.732	0.290	235	0.033	0.004	0.001	0.007
*profile7*	0.712	0.240	**330**	0.080	0.008	0.010	0.008
Alignment-free Features							
*aac*	**0.624**	**0.400**	**1891**	0.033	**0.173**	**0.171**	**0.169**
*Auto_Geary*	0.000	0.310	64	0.000	0.000	0.000	0.000
*Auto_Moran*	0.000	0.320	75	0.000	0.000	0.000	0.000
*Auto_Total*	0.000	**0.370**	1124	0.000	0.000	0.000	0.001
*CTD*	0.408	0.310	1012	**0.070**	**0.134**	**0.133**	**0.137**
*CTD_C*	**0.566**	0.300	**1482**	**0.071**	0.060	0.062	0.066
*CTD_D*	0.407	0.320	**1239**	**0.074**	0.030	0.029	0.033
*CTD_T*	0.529	0.290	**1385**	**0.076**	0.028	0.035	0.036
*fcm*	0.265	0.310	1010	0.012	0.004	0.021	0.021
*2-mers*	0.158	**0.390**	954	0.022	0.003	0.003	0.002
*2-mers_don’t care ps-1*	0.000	0.320	847	0.000	0.000	0.000	0.000
*2-mers_ don’t care ps-2*	0.000	0.310	768	0.001	0.000	0.000	0.000
*2-mers_ don’t care ps-3*	0.000	0.260	772	0.000	0.000	0.000	0.001
*3-mers*	0.078	**0.370**	**1523**	0.064	0.006	0.005	0.006
*3-mers_ don’t care ps-1*	0.000	0.290	600	0.001	0.000	0.000	0.001
*3-mers_ don’t care ps-2*	0.000	0.270	653	0.001	0.000	0.000	0.001
*3-mers_ don’t care ps-3*	0.000	0.270	602	0.002	0.000	0.000	0.001
*length*	**0.507**	**0.400**	**2890**	**0.353**	**0.166**	**0.165**	**0.154**
*nandy*	0.109	0.260	902	0.009	0.000	0.000	0.001
*pseaa10*	0.000	0.240	825	0.000	0.000	0.000	0.001
*pseaa3*	**0.611**	**0.380**	**1397**	0.022	**0.205**	**0.202**	**0.166**
*pseaa4*	**0.609**	**0.380**	**1652**	**0.112**	**0.155**	**0.156**	**0.184**
*QSO_maxlag_30_weight_01*	0.280	0.240	1054	0.075	0.035	0.018	0.020
*QSOCN_maxlag_30*	0	0.250	513	0.001	0.000	0.000	0.001
Alignment-based + Alignment-free Features/Algorithm							
*nw*	**0.789**	0.280	131	**0.786**	**0.382**	**0.373**	**0.374**
*sw*	**0.987**	**0.470**	**646**	0.005	**0.135**	**0.139**	**0.126**
*profile3*	**0.769**	0.280	**271**	0.005	0.098	**0.101**	**0.097**
*profile5*	**0.727**	0.290	**230**	**0.016**	**0.168**	**0.168**	**0.137**
*profile7*	**0.710**	0.260	229	0.004	0.083	**0.084**	**0.126**
*aac*	**0.623**	0.190	**230**	**0.015**	0.073	**0.071**	**0.072**
*Auto_Geary*	0.000	0.300	11	0.000	0.000	0.000	0.000
*Auto_Moran*	0.000	0.270	11	0.000	0.000	0.000	0.000
*Auto_Total*	0.000	**0.510**	147	0.001	0.000	0.000	0.000
*CTD*	0.411	0.360	109	0.005	0.000	0.000	0.000
*CTD_C*	**0.570**	0.340	**204**	**0.039**	0.032	**0.032**	**0.032**
*CTD_D*	0.411	0.390	151	0.009	0.002	0.001	0.001
*CTD_T*	0.531	0.320	164	0.001	0.002	0.003	0.004
*fcm*	0.260	0.300	154	0.005	0.000	0.000	0.001
*2-mers*	0.155	0.200	81	0.003	0.000	0.000	0.000
*2-mers_don’t care ps-1*	0.000	**0.410**	104	0.000	0.000	0.000	0.000
*2-mers_ don’t care ps-2*	0.000	**0.410**	98	0.000	0.000	0.000	0.000
*2-mers_ don’t care ps-3*	0.000	**0.400**	82	0.001	0.000	0.000	0.000
*3-mers*	0.074	0.230	97	**0.010**	0.000	0.000	0.000
*3-mers_ don’t care ps-1*	0.000	0.390	69	0.000	0.000	0.000	0.000
*3-mers_ don’t care ps-2*	0.000	0.340	49	0.001	0.000	0.000	0.000
*3-mers_ don’t care ps-3*	0.000	0.390	59	0.001	0.000	0.000	0.000
*length*	**0.504**	0.230	**231**	**0.059**	0.012	**0.014**	**0.014**
*nandy*	0.113	0.320	101	0.001	0.000	0.000	0.001
*pseaa10*	0.000	0.310	97	0.001	0.000	0.000	0.000
*pseaa3*	**0.613**	0.190	142	0.009	0.006	0.007	0.004
*pseaa4*	**0.610**	0.210	147	0.001	0.005	0.005	**0.009**
*QSO_maxlag_30_weight = 0.1*	0.286	0.270	108	**0.020**	0.001	0.001	0.000
*QSO_maxlag_30*	0.000	0.340	47	0.000	0.000	0.000	0.000

nw: global alignment, sw: local alignment, profile: physicochemical profile from matching regions of aligned sequences at different window sizes (3, 5 and 7), aac: amino acid composition, *pseacc*: *pseudo-amino acid composition at λ = 3,4 and 10, Auto_Geary: Geary’s auto correlation, Auto_Moran: Moran’s auto correlation, Auto_Total: Total auto correlation, fcm: four-color maps, nandy: Nandy’s descriptors, CTD: Composition, Distribution and Transition (Total), CTD_C: Composition, Distribution and Transition (Composition), CTD_D: Composition, Distribution and Transition (Distributions), CTD_T: Composition, Distribution and Transition (Transition), k-mers: 2-mers, 3-mers, spaced words: 2-mers with “don’t care positions” = 1, 2 and 3; 3-mer with “don’t care positions” = 1, 2, 3, QSO: Quasi-Sequence-Order, w = weight factor and maximum lag = 30*

In the alignment-based case, the most important features are those derived from local and global alignments (*sw* and *nw*) besides the physicochemical profile with window size 3 (*profile3*). On the other hand, among the alignment-free features, the amino acid and pseudo amino acid composition of *λ* = 3 and 4, the compositional descriptor (*CTD_C*) along with the length of the sequences turned out to be the most important. When analyzing the alignment-based + alignment-free case, the relevant features are *sw*, *nw*, *profile3*, *profile5*, *profile7*, amino acid composition (*acc*) and *CTD_C*.

## Discussion

### Comparison among supervised classifiers

In the Algorithm Performance experiment, the classification results achieved by our supervised pairwise ortholog detection approach changed slightly when using alignment-based, alignment-free and alignment-based + alignment-free feature combinations. The excellent performance of the alignment-based combinations could be caused by the appropriate selection of the substitution matrixes and gap penalties in relation to the sequence diversity of yeast proteomes [63]. In contrast, alignment-free combinations showed decreasing quality values that may be further improved with other alignment-free pairwise protein features or the inclusion of other similarity measures. In general, the effectiveness of supervised classifiers was not affected by the complexity of datasets when both genomes underwent whole genome duplications (*S. cerevisiae - C. glabrata*), even in the twilight zone. The alignment-based features and the alignment-based + alignment-free combinations along with the Spark imbalanced classification of Random Forest and Decision Trees achieved better effectiveness, as well as faster ortholog pair detection times even in such a complex dataset. The inclusion of different pairwise similarity measures in the decision system may prevent the algorithm from missing gene/protein pair relationships during the classification process.

This study corroborates the results of our previous paper [36] in the sense that supervised classifiers managing the extreme ortholog pair class imbalance outperform the original classifiers without class imbalance management. In addition, the success of the RUS pre-processing approach is accompanied by a considerable reduction in execution time. Specifically, ROS and RUS Random Forest and Decision Trees Spark algorithms showed prominent quality values in ortholog classification, likewise in the twilight zone. This simple decision tree surpassed tree ensembles in Random Forest even when alignment-free features had just been used, and its performance was improved with the alignment-based + alignment-free combination in the *ScerCgla* dataset; which contains “traps” for ortholog prediction algorithms. Such “traps” consist of the paralogs originated from the whole genome duplication event that the genomes *S. cerevisiae* and *C. glabrata* underwent, subsequently the loss of many of these paralogs provides confusion to the ortholog prediction algorithms [27].

When dealing with Spark Random Forest classifiers, small differences were shown when applying different Impurity metrics, namely entropy and Gini. Although entropy led to better results in the alignment-free and the alignment-based + alignment-free cases, Gini could be preferable as due to its efficiency, i.e. there is no need to compute the logarithmic expression. In this sense, future studies should be oriented towards the tuning of other parameters’ values, namely pre-processing policies, number of trees, or maximum depth, as these may allow significant differences to be obtained in terms of both efficiency and accuracy.

Regarding the comparison between Spark and Hadoop Random Forest implementations, the former showed a significant reduction in execution time while increasing the quality of classification. Efficiency is due to the capabilities of Spark when embedding data into memory to avoid disc overhead, whereas classification performance is achieved by a smart design of the learning procedure where the model is built iteratively using all available data [64].

### Comparison of feature combinations

From the feature importance evaluation in supervised classifiers we can conclude that alignment-based features should continue to be of much importance for ortholog detection, mainly when the local alignment is combined with the global alignment and the physicochemical profile features are derived from matching regions of pairwise aligned proteins. The exclusion of synteny (membership of a protein pair to locally collinear blocks (LCBs)) among the alignment-based similarity measures did not affect the classification performance in relation to our previous report [36]**.** However, when alignment-based features were combined with alignment-free features selected in the study the results slightly improved in some datasets, similarly in the twilight zone. This is a new motivation for further research aimed at analysing the inclusion of new alignment-free features or the tuning of parameter values.

The results obtained so far emphasize the importance of the local sequence similarity to detect protein functional similarity so that most of the classical ortholog detection methods start with BLAST alignments intrinsically based on the identification of query substrings or *k*-letter words [2, 38, 65, 66]. On the other hand, the alignment-free approach for ortholog detection proposed by [38] works on the hypothesis that the higher the number of common *k*-mers between two sequences, the higher may be their functional similarity. In a similar way, the compositional alignment-free features such as amino acid composition, pseudo-amino acid composition and composition, transition and distribution also seem to be highly related with the ortholog concept since they have been useful for functional similarity detection.

### Comparison of supervised versus unsupervised classifiers

The success of the supervised algorithms might have been obtained from the combination of several alignment-based pairwise features like global and local alignment scores and the physicochemical profiles at different window sizes, as well as from the recent incorporation of alignment-free measures together with the training from curated datasets. By combining global and local alignment similarities, we have considered structural and functional protein similarities, respectively. These protein features have been complemented with physicochemical and alignment-free information in order to cope with homology detection pitfalls caused by significant matches of short sequences, remote homology, convergent evolution and other evolutionary and genetic events. Precisely, the alignment-based + alignment-free feature combination together with all the Spark and the pre-processing benefits allowed Decision Trees to detect remote orthologs at higher success rate in the complex *ScerCgla* dataset, which contains “traps” for ortholog detection. Conversely, the lesser classification quality values of RBH, RSD and OMA, mostly of RBH, can be explained by their only sequence similarity approach [66], although BLAST parameter values have been tuned following the recommendation in [65]. However, the remarkable stable performance of RSD (α = 0.8 and E-value = 1e-05) has been significant, even within the twilight zone of two proteome pairs (*CglaKlac* and *KlacKwal*) where it could detect a higher number of orthologs than our supervised proposals with just alignment-free features. This achievement might be the result of the RSD intrinsic combination of similarity and evolution distance information [11].

It is worthy to mention that pure alignment-free supervised classifiers showed similar performance than the RSD algorithm for ortholog detection; and when alignment-free features are incorporated into Spark Decision Trees with imbalance management, a higher success rate (98.71%) was achieved within the twilight zone of the more complex yeast proteome pair (*ScerCgla*) which underwent a whole genome duplication and subsequent differential losses of gene duplicates.

The experiments carried out corroborate Kuzniar’s farsighted criteria that algorithms incorporating various sources of knowledge should yield promising results in ortholog detection [2]. However, he pointed out that a scalable, fully automated procedure to infer orthologs across genomes of all kingdoms of life remains an elusive goal. For this reason, our proposals should be thoroughly tested with other benchmark eukaryotic genomes/proteomes in order to extend its usefulness.

## Conclusions

The extension of previous experiments to different yeast species from the *Saccharomycete* class corroborated the validity of our big data supervised classification approach that manages data imbalance for ortholog detection. The top-ranked Spark algorithms (Random Forest and Decision Trees) managing the imbalanced rate between orthologs and non-orthologs have surpassed the Hadoop MapReduce Random Forest classifiers with the best results in our previous work, considering both the quality of classification and the execution time. Although the introduction of alignment-free pairwise features into tree-based supervised models did not significantly improve the classification rates achieved with several alignment-based similarity measures, it was shown that some compositional alignment-free features might have positively contributed to ortholog detection, especially to identify orthologs at the twilight zone. The introduction of the alignment-free features in ortholog detection is an open field that we will keep exploring in future research.

## Additional file

Additional file 1: Proteome fasta files for the following yeast species: *S. cerevisiae, C. glabrata, K. waltii* and *K lactis*. (ZIP 5844 kb)
